# The replication of Bangladeshi H9N2 avian influenza viruses carrying genes from H7N3 in mammals

**DOI:** 10.1038/emi.2016.29

**Published:** 2016-04-20

**Authors:** Karthik K Shanmuganatham, Jeremy C Jones, Bindumadhav M Marathe, Mohammed M Feeroz, Lisa Jones-Engel, David Walker, Jasmine Turner, S M Rabiul Alam, M Kamrul Hasan, Sharmin Akhtar, Patrick Seiler, Pamela McKenzie, Scott Krauss, Richard J Webby, Robert G Webster

**Affiliations:** 1 Department of Infectious Diseases, St Jude Children's Research Hospital, Memphis, TN 38105, USA; 2 Department of Zoology, Jahangirnagar University, Dhaka 1342, Bangladesh; 3 National Primate Research Center University of Washington, Seattle, WA 98195-5502, USA

**Keywords:** Bangladesh/epidemiology, H9N2 subtype/classification, influenza virus/genetics, viral characterization, virus biology, risk assessment, viral pathogenesis, zoonosis

## Abstract

H9N2 avian influenza viruses are continuously monitored by the World Health Organization because they are endemic; they continually reassort with H5N1, H7N9 and H10N8 viruses; and they periodically cause human infections. We characterized H9N2 influenza viruses carrying internal genes from highly pathogenic H7N3 viruses, which were isolated from chickens or quail from live-bird markets in Bangladesh between 2010 and 2013. All of the H9N2 viruses used in this study carried mammalian host-specific mutations. We studied their replication kinetics in normal human bronchoepithelial cells and swine tracheal and lung explants, which exhibit many features of the mammalian airway epithelium and serve as a mammalian host model. All H9N2 viruses replicated to moderate-to-high titers in the normal human bronchoepithelial cells and swine lung explants, but replication was limited in the swine tracheal explants. In Balb/c mice, the H9N2 viruses were nonlethal, replicated to moderately high titers and the infection was confined to the lungs. In the ferret model of human influenza infection and transmission, H9N2 viruses possessing the Q226L substitution in hemagglutinin replicated well without clinical signs and spread via direct contact but not by aerosol. None of the H9N2 viruses tested were resistant to the neuraminidase inhibitors. Our study shows that the Bangladeshi H9N2 viruses have the potential to infect humans and highlights the importance of monitoring and characterizing this influenza subtype to better understand the potential risk these viruses pose to humans.

## INTRODUCTION

Influenza is a serious ongoing threat to human and animal health. Every year, thousands of people are infected with seasonal influenza. In addition, they may be exposed to avian (H5, H6, H7, H9, and H10) and swine (H1 and H3) influenza subtypes.^1^ During past human influenza pandemics, the causative virus subtypes originated either partially or entirely from avian influenza viruses that had crossed the species barrier.^2, 3, 4, 5^ Within the past decade, avian influenza subtypes H5N1, H7N9, and H9N2 have become endemic in poultry in Eurasia and are the leading candidates that have the potential to transmit to humans and cause lethal infection.^6, 7^ Each of these influenza subtypes has domestic poultry as an intermediate host and is predominantly isolated from live-bird markets, which are a proven risk factor for zoonotic transmission between birds and humans. Highly pathogenic H5N1, human H7N9, and more recently H10N8 possess the internal gene cassette from poultry H9N2 viruses; thus, it has been hypothesized that poultry H9N2 virus, in addition to retaining the ability to infect humans, might enable H5, H7 and H10 subtypes to infect and transmit to humans.

In North America, the H9N2 subtype was first isolated from turkeys in 1966.^8^ Distinguishable lineages of H9N2 have become enzootic in land-based poultry in Asia, Africa and the Middle East.^9, 10, 11, 12^ In poultry, H9N2 infection is usually asymptomatic, with little to no mortality and decreased egg production.^13^ Experimentally, H9N2 virus infects mice and has been isolated from pigs and dogs in China.^10, 14, 15, 16^ H9N2 has also sporadically infected humans without causing mortality. To date, 11 human H9N2 infections have been reported in China; two cases have been reported in Bangladesh and three in Egypt.^15, 16, 17, 18, 19^ Recent phylogenetic classification of the H9N2 shows that this subtype has evolved into 23 distinct clades with five clade-specific outliers.^20^ The G1-like lineage represented by A/quail/Hong Kong/G1/1997 and the Y280-like lineage represented by A/chicken/Hong Kong/G9/1997 are the two most predominant progenitor lineages of the H9N2 viruses currently circulating in China, the Middle East and Southeast Asia.^12, 21, 22^ Surveillance reports from the Middle East, India and Pakistan, show that H9N2 viruses circulating in those areas are genetically related to the G1 clade but have evolved into distinct subclades.^23^

Surveillance studies from Bangladesh have shown that H9N2 is endemic in chickens and quail and that live-bird markets serve as an epicenter for infection and transmission.^24, 25^ Bangladeshi H9N2 viruses can be traced back to the G1 lineage.^23^ Since their introduction during the early 2000s, these H9N2 viruses have evolved tremendously via intra- and intersubtype reassortment and genetic drift to become unique and distinct from the prototype G1 viruses.^23^ Bangladeshi H9N2 avian influenza viruses' hemagglutinin (HA) belongs to the Iranian H9N2 lineage (Mideast_B); its neuraminidase (NA) and polymerase basic 2 (*PB2*) genes are from the Dubai H9N2 lineage (Mideast_C Dubai); and the nonstructured protein (NS), nucleocapsid protein (NP), matrix (M), polymerase acidic (*PA*) and polymerase *PB1* genes are from highly pathogenic avian influenza (HPAI) H7N3 viruses originating in Pakistan.^23^ In addition, Bangladeshi H9N2 viruses have acquired numerous molecular makers throughout the genome that facilitate host-range transmission from avian species to humans.^23^

Few studies have examined the replication potential and transmissibility of avian H9N2 viruses to mammals, and almost all of those have focused on G1 viruses isolated from China. To date, very little information is available on the pathogenesis, virulence and transmission of Bangladeshi H9N2 viruses. To close this gap, we determined the potential of Bangladeshi H9N2 viruses carrying H7N3 internal genes to replicate in mammals. We modeled the replication and pathogenesis of these viruses in *ex vivo* cultures of human cells and swine tissues and tested the viral susceptibility and transmission in mice and ferrets.

## MATERIALS AND METHODS

### Ethics statement

All animal studies were approved by the St Jude Children's Research Hospital Animal Care and Use Committee and were performed in compliance with the policies of the National Institutes of Health and the Animal Welfare Act. All experiments were carried out by trained personnel working in a United States department of agriculture–inspected animal biosafety level 3+ animal facility.

### Viruses

The H9N2 viruses used in this study (Table 1) were isolated during our ongoing avian influenza surveillance in live-bird markets in Bangladesh. The viruses were chosen because they are representative of the molecular changes that have occurred in the genomes of H9N2 viruses circulating in Bangladesh. All H9N2 influenza viruses were propagated and titrated in the allantoic cavities of 10-day-old embryonated chicken eggs at 35 °C for 48 h. Virus titers were determined by injecting 100 μL of 10-fold dilutions of virus into the allantoic cavities and then calculating the 50% egg infectious dose (EID_50_) according to the method of Reed and Muench.^26^

### Cell culture

Madin-Darby canine kidney (MDCK) cells (ATCC, Manassas, VA, USA) were serially passaged and maintained in culture in modified Eagle's medium (CellGro, Herndon, VA, USA) supplemented with L-glutamine (2 mM), 5% fetal bovine serum and antibiotics at 37 °C with 5% CO_2_. Normal human bronchial epithelial (NHBE) cells (Lonza, Walkersville, MD, USA) from two healthy male donors (two and four years old) were expanded, cryopreserved and maintained in culture in an air/liquid interface (ALI) system, as previously described.^27, 28^ Briefly, cells were plated in 0.33 cm^2^ transwell inserts (Corning, Corning, NY, USA) and allowed to differentiate. An ALI was established when the cells reached 98%–100% confluence. Media from the apical surface of the cells was removed, and the cells were exposed to a humidified 95% air/5% CO_2_ environment. The basolateral media or the ALI media, which was supplemented with SingleQuot growth factors (Lonza), was changed every 48 h for a minimum of six weeks. During every media change, the apical surface of the cells was washed with sterile phosphate-buffered saline (PBS) to remove mucus.

### Swine tracheal and lung explants

The replication properties of the Bangladeshi H9N2 viruses were tested in juvenile swine tracheal and lung explants. Juvenile trachea and lung were obtained from female piglets (three to four weeks, Midwest Research Swine, Gibbon, MN, USA). Due to logistical limitation and availability of juvenile respiratory tissue, we conducted only one independent experiment in duplicates. Both trachea and lungs were harvested within 1 h of killing and processed as described.^29, 30^ Briefly, the isolated tracheas were washed with PBS containing penicillin–streptomycin–amphotericin B (Sigma, St Louis, MO, USA), split open lengthwise and pinned to a dissection board. Intact tracheal biopsy explants were obtained using a disposable 5-mm biopsy punch. The isolated explants were then incubated at 37 °C in 5% CO_2_ at an ALI in transwell inserts similar to conditions used to grow the NHBE cells for at least 24 h before infection, with the following exception: the basolateral medium was changed hourly for the first 4 h.

For the lung tissue, we adopted and modified the method described by Chan *et al.*^29^ The isolated lungs were first perfused with cold transport inserted through the bronchiole, perfused again with 1% low-melt agarose in PBS (Sigma), surrounded with icepacks until the agar solidified. The agarose-perfused lungs were then dissected into smaller tissue pieces (~2 × 1 × 1 cm size) and embedded in 4% agarose. Thin slices were prepared from embedded lungs with a 1-mm microtome blade and explants (5 mm diameter) were prepared with biopsy punches handled similarly to the tracheal explants.

### H9N2 infection of NHBE cells and swine tracheal and lung explants

Prior to H9N2 infection, the apical surfaces of the NHBE cells or explants were washed with PBS pre-warmed to 37 °C, followed by equilibration with bronchial epithelial cell basal medium (Lonza) infection medium containing 0.5% bovine serum albumin at 37 °C for 30 min. Multiplicity of infection of 0.01 for NHBE cells or 10^6^ EID_50_ units per explant in 100 μL of infection media was prepared for each virus and added to the apical surface of the tissues. Viruses were allowed to adsorb at 37 °C; after 1 h, the virus dilutions were removed by aspiration, and the apical surface was washed with PBS (pH 2.2) × 3 and PBS (pH7.0) × 3 to remove unbound virus and reduce background. Tissues were transferred to a new well containing fresh ALI medium with 1 × antibiotic/antimycotic and incubated at 37 °C. Samples were collected from the apical surface at 28, 48, 72 and 96 h. At the indicated time point, 300 μL infection medium was added to the apical surface for 30 min at 37 °C and then removed. Viral titers were determined immediately on MDCKs, with 50% tissue culture infectious dose (TCID_50_) determined using the method of Reed and Muench.^26^ Both the NHBE cell and swine-explant data are representative of combined data from two independent tests of three inserts per virus group.

### H9N2 infections in mice

Six-week-old Balb/c mice (Charles River, Wilmington, MA, USA) were lightly anesthetized with isoflurane (Baxter Healthcare, Deerfield, IL, USA) and intranasally administered 10^6^ EID_50_ units of virus in a 30-μL volume. Mice (*n*=5/virus group) were monitored daily for morbidity (that is, weight loss, lethargy, coat conditions) and mortality. Two mice were killed at three and five days post infection (d.p.i.), respectively, and a section of lung was removed and homogenized in 1 mL PBS. Viral titer (TCID_50_) values were determined as previously described. At 14 d.p.i., blood from each of the experimental groups was collected and subjected to the hemagglutination inhibition (HI) assay^31^ with chicken red blood cells and homologous H9N2 viruses to test for seroconversion.

### H9N2 infections in ferrets

3- to 6-month-old male ferrets (Triple F Farms, Sayre, PA), which were sero-negative for influenza A (H1 and H3 subtypes) and B, were used in the experiment. Two Bangladeshi H9N2 viruses were tested alongside the control H9N2 virus. For each virus group, three inoculated donors and three direct contacts were housed in the same cage, and three aerosol contacts were housed in an adjacent cage separated by a permeable barrier that allowed free airflow but not physical contact. Prior to infection, all of the ferrets were weighed, and their body temperatures were obtained. On the day of infection, donor ferrets (*n*=3 for each virus group) were anesthetized using isoflurane, and 10^6^ EID_50_ units of avian influenza virus in 1 mL solution were administrated intranasally. Twenty-four hours later, direct contacts (*n*=3 for each virus group) and aerosol contacts (*n*=3 for each virus group) were introduced into their respective cages. Every 24 h, all ferrets' temperatures and weights were recorded. At 2, 4, 6, 8 and 10 d.p.i., the ferrets were sedated with ketamine (25 mg/kg body weight),^32, 33^ and nasal washes were collected with 1 mL sterile PBS. The viral titers of the nasal washes were determined in MDCK cells, and TCID_50_ values were determined as previously described. At 16 d.p.i., blood was collected from all ferrets, and subjected to the HI assay^31^ with chicken red blood cells and homologous H9N2 viruses to test for seroconversion.

### Susceptibility of H9N2 viruses to NA inhibitors

The NA inhibitors (NAIs) oseltamivir carboxylate (oseltamivir; Hoffmann-La Roche, Basel, Switzerland), zanamivir (GlaxoSmithKline, Research Triangle Park, NC, USA) and peramivir (BioCryst Pharmaceuticals, Birmingham, AL, USA) were used to test the sensitivity of the Bangladeshi H9N2 viruses. The NA activity was determined by a modified fluorometric assay using the fluorogenic substrate 2′-(4-methylumberlliferyl)-α-d-N-acetylneuraminic acid (Sigma-Aldrich, St Louis, MO, USA) as described previously.^30, 34, 35^ A/Fukui/20/2004 (E119) (H3N2) (wild-type virus) and A/Fukui/45/2004 (E119V) (H3N2) were used as reference viruses and are part of the World Health Organization's NAI-susceptibility panel.

### Statistical analyses

Statistical analysis and graph were done using GraphPad Prism six software (La Jolla, CA, USA) and values were expressed as the mean±SD of the mean. A two-way analysis of variance test was used to determine the statistical significance for each individual H9N2 viruses that were used to infect NHBE cells. To determine the statistical significance between input inoculum and productive viral replication an analysis of variance comparison between 2 and 72 h post infection within the same virus was used. For both cell types a *P*-value ⩽0.05 was considered significant.

## RESULTS

### Virus selection

To assess the biologic properties of the Bangladeshi H9N2 viruses and their risk to mammals, we selected nine representative isolates (Table 1) from our ongoing avian influenza surveillance project in Bangladeshi live-bird markets. The viruses were previously characterized molecularly and phylogenetically,^23, 25^ and they are representative of the year, location and host species from which they were isolated. The nine H9N2 isolates were isolated during 2010–2013, and their genotypes are representative of H9N2 viruses that are circulating in chickens or quail. All of the viruses carry at least three internal genes (*NS*, *PA* and *PB1*) from HPAI subtype H7N3 from Pakistan.^25, 36^ These selected viruses were screened for other influenza subtypes, including HPAI H5N1, and for Newcastle disease virus. One isolate, A/quail/Bangladesh/19462/13, was a H9N2/H5N1 mixture, and the H9N2 was isolated from the mixture by two simultaneous egg-neutralization passages in the presence of H5N1 antisera. The absence of HPAI H5N1 was confirmed using H5-specific real-time probes and conventional PCR primers.

### Molecular analyses of Bangladeshi H9N2 viruses

All of the Bangladeshi H9N2 isolates used in this study had molecular changes throughout the viral genome that are associated with host-range specificity and pathogenesis (Table 2). Most substitutions were identified in the viral surface glycoprotein HA, which is involved in viral entry into the host cell. In the HA, three important substitutions (183H, 226L, and 391K) were located in the receptor-binding site of all the H9N2 isolates tested, except A/environment/Bangladesh/10306 (quail) H9N2, which still had the avian host-specific residue at position 226(Q). The Q226L shift is very important in the affinity switch from avian-type to human-type sialic acid receptors.^37^ Six of the seven H9N2 isolates used in this study had the KSKR motif in the HA1/HA2 cleavage site, and this motif has been predominately identified in the Bangladeshi H9N2 isolates.^25^ In the NA, none of the isolates used in this study had both the E119V and R292K substitutions, which are associated with resistance to antiviral drugs such as oseltamivir and zanamivir. In the internal genes, numerous human host-specific changes were identified in PA, PB1, M, NP and NS (Table 2). In each of the H9N2 isolates tested, most of the mammalian host-specific substitutions were fixed/permanent and were representative of the genome of the H9N2 currently circulating in Bangladesh.^25, 36^ The substitutions were predominately in PB1 (L13P), NP (E372D), M1 (V15I), and M2 (L55F and S31N) and were associated with amantadine resistance (Table 2). In the NS1 protein, all seven of the H9N2 isolates carried the E227K substitution in the C-terminal region, which modulates pathogenicity in avian influenza viruses.^38^ None of the isolates carried the human-adaptation marker E627K in the *PB2* gene.

### H9N2 replication in primary differentiated normal human bronchoepithelial cells

**Figure 1:**
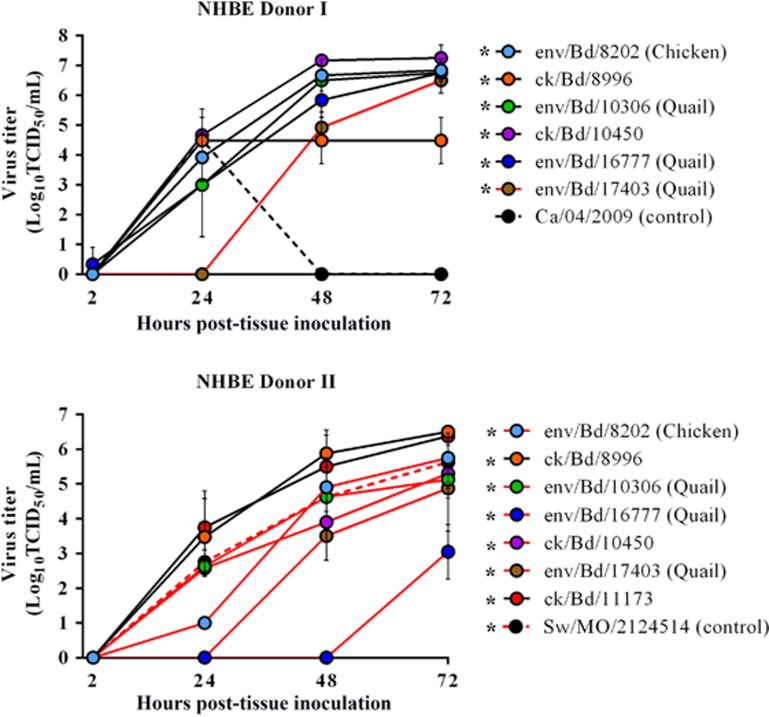
Replication kinetics of avian H9N2 viruses in normal human bronchial epithelial cell. Differentiated NHBE cell (*n*=2–3 inserts/virus group) from two distinct human donors (two and four years of age) were inoculated with representative H9N2 viruses (MOI 0.01) and virus titers were determined at indicated time points. Delayed replication <3 log_10_ TCID_50_/mL at 24 h.p.i.) is indicated by red lines. Mammalian virus controls are indicated by dotted lines. Data are presented as mean titer ±s.d. Statistical significance of replication (comparing 2 h.p.i. to 72 h.p.i. values) was determined by two-way ANOVA. *P<0. analysis of variance, ANOVA; multiplicity of infection, MOI; normal human bronchial epithelial, NHBE; tissue culture infectious dose, TCID.

Primary well-differentiated NHBE cells are morphologically and physiologically similar to cells in the human respiratory tract.^39^ Therefore, we used this cell line as an *in vitro* model system to assess if the Bangladeshi avian H9N2 viruses possess the ability to infect and productively replicate in human cells. The apical surfaces of H9N2 inoculated (multiplicity of infection of 0.01) NHBE cells were sampled for virus replication, 2–72 h post infection (h.p.i.). All of the Bangladesh H9N2 viruses tested replicated in both the NHBE donors from a moderately low (2.5 Log_10_ TCID_50_/mL) to high titers (7 Log_10_ TCID_50_/mL) with a detectable difference in replication kinetics between the chicken and quail viruses as well as between the donors. None of the Bangladeshi viruses showed qualitative cytopathic effect in the NHBE cells. In both the donors cells within 24 h.p.i., the chicken H9N2 viruses with the exception of env/Bangladesh/8202 in NHBE donor 1 had viral titers (1–3.8 log_10_ TCID_50_/mL) similar to or higher than the mammalian control viruses (A/swine/Missouri/ 2124514/2006 (H2N3) and A/Ca/04/2009 (H1N1) virus (2.8 log_10_ and (1–3.8 log_10_ TCID_50_/mL, 4.5 log_10_ TCID_50_/mL, respectively); Figure 1) and viral titers peaking from 5.9 log_10_ TCID_50_/mL at 48 h.p.i. to 6.5 log_10_ TCID_50_/mL at 72 h.p.i., (Figure 1). Replication for one of the quail H9N2 virus (A/env/Bangladesh/16777/2012) was only delayed in one donor and was not detected until 72 h.p.i. For the other quail virus (A/env/Bangladesh/17403/2012) replication was delayed in both the donors and did not reach comparable titer (3.5–5.6 log_10_ TCID_50_/mL) until after 48 h.p.i. (Figure 1).

### H9N2 replication in swine tracheal and lung explants

Swine serve as an intermediate host/mixing vessel for reassortment of avian influenza viruses. Chinese lineage H9N2 has been shown to replicate in pigs^40^ therefore, to determine whether Bangladesh lineage H9N2 are capable of productive replication, we utilized an *ex vivo* model of tissue explants prepared from swine respiratory tract. Previous studies have used swine tracheal and lung explants to assess the replication potential of avian influenza viruses in swine.^41, 42, 43, 44^ Each of the H9N2 viruses tested caused a productive infection and stably replicated to high titers in the lung explants, compared with that in the tracheal explants. Viral replication was slow for four (A/env/Bd/8202, A/ck/Bd/8996, A/env/Bd/16777 and A/env/Bd/17403) of the seven H9N2 viruses tested in trachea. Moderately low peak titer of 2.5 log_10_ TCID_50_/mL was mostly observed by 24 h.p.i. with no increase in viral titers till 72 h.p.i. (Table 3). Two chicken H9N2 viruses (A/ck/Bd/10450 and A/ck/Bd/11173) replicated to moderately high titer (4.3–4.5 Log_10_ TCID_50_/mL) by 72 h.p.i. (Table 3). One quail H9N2 virus (A/env/Bd/10306) failed to establish a productive infection (Table 3). As expected, the positive control, A/swine/Missouri/2124514/2006 (H2N3), replicated to high titer (6.3 log_10_ TCID_50_/mL) at 48 h.p.i., whereas the negative control, A/Dk/NJ/872–27/78 (H2N2), had no detectable titers, after 72 h.p.i. (Table 3). When compared with the trachea, in the lungs the chicken H9N2 viruses (A/env/Bd/8202, A/ck/Bd/8996A/ck/Bd/10450 and A/ck/Bd/11173) replicated to moderately high titer of 4.5 to 5.5 log_10_ TCID_50_/mL by 48 h.p.i. Two of the three quail H9N2 viruses (env/Bd/10306 and env Bd/17403) showed delayed replication kinetics where viral titers were only observed only after 48 h.p.i. As expected, the positive control, A/swine/Missouri/2124514/ 2006 (H2N3), replicated to a high titer (6.3Log_10_ TCID_50_/mL) at 48 h.p.i., whereas the negative control, A/Dk/NJ/872-27/78 (H2N2), had no detectable titers, after 72 h.p.i. (Table 3).

In summary, Bangladeshi H9N2 viruses preferentially replicate in swine lungs with less preference to trachea, which is likely attributed to the distribution of the α-2, 3 and the α-2, 6 sialic acid receptors in the pig's respiratory organs.^45^

### H9N2 infection in mice

Mice can serve as an initial mammalian model of influenza infection and viral replication in mice without adaptation is a high risk factor of virulence in humans and animals.; thus, we conducted a preliminary experiment with six H9N2 viruses (Table 4) in which we inoculated 6-week-old BALB/c mice with 10^6.8^ EID_50_/mL units of a virus in 30 μL PBS (*n*=5 mice per viral dose per virus isolate). Morbidity and mortality were monitored daily for 14 d.p.i. To determine the virus titer in the lungs, we infected a second group of BALB/c mice (*n*=2 for day 3 and *n*=2 for day 5, *n*=4 mice per virus isolate). Uninfected (PBS-inoculated, *n*=5) and A/Puerto Rico/8/34 (*n*=5) (PR8)–inoculated mice served as controls. None of the BALB/c mice died or displayed clinical disease during the entire course of the study. As expected, control PR8-inoculated mice either lost more than 30% of their baseline body weight and were euthanized or were dead 4–5 d.p.i. (Table 4). Five of the six avian H9N2 viruses that were tested productively replicated in the lungs of the Balb/c mice with viral shedding lasting at least 5 d.p.i. and seroconversion at 14 d.p.i. Five of the six H9N2 viruses tested did not cause significant weight loss (>10%) post infection (Table 4), but mice infected with the quail H9N2 virus (env/Bd/10306) showed a 15% weight loss 3–5 d.p.i. and recovered thereafter (Table 4). Viral titers determined in the lungs showed that the titers for most of the H9N2 viruses peaked at 3 d.p.i. (~5 log_10_ TCID_50_/mL; Table 4). For one of the quail viruses (env/Bd/10306), titers were at the limit of detection (1 log_10_ TCID_50_/mL) on both 3 and 5 d.p.i. (Table 4). HI assays done with the homologous viruses at 14 d.p.i. terminal bleeds showed that all mice, with the exception of a single mouse in the quail H9N2 virus group (env/Bd/10306), had seroconverted (Table 4). Overall, Bangladeshi H9N2 viruses caused subclinical infections in the mice without causing any outward manifestation of influenza infection but still demonstrated productive replication capacity in the lungs.

### H9N2 infection in ferrets

Ferret lung physiology is very similar to that of humans, and ferrets exhibit several clinical signs associated with human influenza infection. Thus, the ferret animal model is the current gold standard for modeling human influenza pathogenesis and transmissibility. To evaluate the infection potential of the Bangladeshi H9N2 viruses, we undertook a preliminary study involving two distinct Bangladeshi H9N2 viruses (env/Bd/10306 and ck/Bd/10450) with differing molecular genotypes harboring various mammalian host-specific substitutions throughout their genomes, especially in the receptor-binding site of the HA glycoprotein (Table 2). We infected donor ferrets with either env/Bd/10306 or ck/Bd/10450 and used A/ck/Pakistan (NARC)/1624/2005 as a control. In addition, to assess the transmissibility of the viruses, we introduced ferrets as cage mates (direct contact) or placed them in adjacent cages (aerosol contact) at 24 h.p.i.

None of the donor ferrets exhibited clinical signs (for example, fever, lethargy, weight loss or nasal discharge). Nasal titers from the donor ferrets from both H9N2 test groups showed that a productive infection was established within 2 d.p.i., and the animals were shedding virus nasally for at least 6 d.p.i. (Table 5) Between the two H9N2 viruses, donors infected with A/chicken/Bd/10450/2011 shed more virus (~6 Log_10_ TCID_50_/mL) than did the donor ferrets infected with env/Bd/10306 (~4 log_10_ TCID_50_/mL) at 2 d.p.i. (Table 5). Despite a productive viral infection, direct-contact transmission was seen only in ferrets infected with ck/Bd/10450 (Table 5). In that group, only two of three ferrets were infected, and one ferret shed virus for only 1 day (5 d.p.i., 5.8 log_10_ TCID_50_/mL), and the other ferret shed much smaller viral titers than did the donor (2.5–4 Log_10_ TCID_50_/mL) (Table 5). No aerosol transmission was observed in either virus group (Table 5). All ferrets in the control group (ck/Pakistan (NARC)/1624) had viral titers in the nasal washes but did not produce any clinical signs (Table 5). All ferrets that got infected (donors and direct contact) and shed virus also seroconverted (Table 5). Seroconversion of the donor ferrets infected with env/Bd/10306 was eightfold less than that in the donor ferrets infected with ck/Bd/10450 (HI 160 vs HI 1280) (Table 5). All remaining ferrets that were not infected remained seronegative (Table 5).

When the ferret nasal washes were compared with viral titers obtained from NHBE cells and swine explants, both the Bangladeshi H9N2 viruses had similar replication titers (Tables 3, 4, and 6) *in vivo* and *in vitro*. On the basis of this preliminary study, it appears that the Bangladeshi H9N2 viruses are capable of causing a productive subclinical infection in ferrets and might have the potential to transmit to animals in very close contact and under ideal conditions. However, none of the H9N2 viruses appeared to currently possess the potential for aerosol transmission.

### Antiviral susceptibility

To test whether the Bangladeshi H9N2 viruses are sensitive to the currently approved antiviral drugs, we examined the susceptibility these H9N2 viruses to the NAIs oseltamivir, zanamivir and peramivir. All six H9N2 viruses tested were fully susceptible to the three NAIs, with mean IC_50_ values ranging from 0.13 to 0.69 nM (Table 6). These IC_50_ values are similar to the NAI-susceptible control A/Fuk/20/04 (H3N2, World Health Organization's NAI-susceptibility panel), as well as the NAI-susceptible human H1N1 and H3N2 influenza A viruses.^46^

## DISCUSSION

H9N2 avian influenza viruses are endemic in terrestrial poultry and have evolved into many permanent lineages (G1, G9, Y280 and Ck/Bei-like) in Asia. The Bangladeshi H9N2 viruses used in this study belong to a distinct G1 sublineage containing multiple internal genes from HPAI H7N3 and harbor numerous mammalian host-specificity markers. Despite these numerous genotypic risk factors few studies have addressed the ability of these unique Bangladeshi H9N2 viruses to infect mammals. In this study, we investigated this issue by testing the viruses in various *in vitro* and *in vivo* mammalian model systems. We showed that the Bangladeshi H9N2 viruses can productively infect and replicate in NHBE cells, swine respiratory tissue, mice and ferrets after experimental inoculation. The H9N2 viruses replicated without causing any qualitative cytopathic effects in the *in vitro* model systems and replicated primarily in the respiratory tissue of mice and ferrets without inducing clinical signs of infection and were susceptible to currently approved NAIs. The lung appears to be the preferred site of replication for the H9N2 viruses.

In the NHBE cells and swine tissue explants, the H9N2 viruses that we tested replicated to moderate-to-high titers. Previous studies in NHBE cells have shown that replication of influenza viruses between 8 and 24 h.p.i. directly correlates with infection in humans,^47^ but the higher replication titers that we observed were seen only after 48 h.p.i. Thus, the Bangladeshi H9N2 viruses can replicate in NHBE cells, despite lesser prevalence of the α-2,3 sialic acid linkages in the human cells.^39^ Studies have shown that H9N2 viruses harboring the Q226L mutation in HA display a 100-fold higher tropism for human airway epithelium when compared with H9N2 viruses with only the Q226 substitution.^37^ With the exception of the quail isolate (env/Bd/10306), all of the H9N2 isolates tested in this study had the Q226L mutation. In addition, all of the H9N2 viruses had the following mammalian host–specific mutations: HA (183H, 226L 391K), M1 (15I), M2 (55F and the drug-resistance marker 31N), NS (227K), NP (109V, 214K, and 372D) and PB1 (13P). This may explain why Bangladeshi H9N2 viruses may have recognized the α-2,6-linked sialic acid receptors present in the NHBE cells and were able to replicate in them. Studies of human H5N1 infection^48^ and swine-origin H1N1 infections^49, 50^ have shown that molecular changes are required for adaptation to new host species.^51^ In addition, HA with modified glycans can recognize the α-2,3 sialic acid residue with decreased specificity and increased affinity,^52^ and a single amino acid substitution in the HA abrogates biding to the sialic acid residue.^53^ Therefore, understanding the molecular features of these avian H9N2 viruses is crucial to understanding their host-range switch.

Our swine explant data showed that H9N2 viruses replicate to less titers in juvenile swine tracheal cells, but the lung titers were similar to that of the swine control virus. Studies have shown that A/Quail/HK/G1/97 (H9N2), the progenitor of the Bangladeshi H9N2 viruses and the donor of the internal genes of HPAI H5 that infected humans in 1997, had similarly limited replication properties in the pig trachea compared to that in the lungs.^29, 41^ Viruses that switch tropism from avian type to mammalian type also have tropism for the lower respiratory tract, as seen in the Bangladeshi H9N2 viruses.^54^ This result is also seen in α-2,3-binding H5N1 and α-2,6-binding H9N2 avian viruses.^41^ Alternatively host and viral process involved during an infection could influence the viral replication kinetics in pig trachea. Host defenses like the cell wall, mucosal surface and host factors that bind to virion components may prevent H9N2 viral replication. In addition, even if virus binds to the cell receptor, restriction to replication may also occur at different stages of the viral infection cycles. The host interferon responses during the initial stages of infection may protect the cell against influenza infection. The other factor that might influence replication in pig trachea is the genetic composition of the H9N2 viruses, that is, the current Bangladeshi H9N2 viruses may be poorly adapted and that mutations that are occurring within its genome are not optimized for adaption in the new host and may require further more compensatory mutation to attain replication competence in mammalian tissue. The preferential replication of the H9N2 virus in lungs, compared with that in the trachea, may be restrictive for these avian viruses to cross the species barrier. If the viruses are unable to establish a productive infection in the upper respiratory tract, then they are most likely to be cleared from the animal before they reach the lungs. This is consistent with the finding that H9N2 infections of man and swine are very sporadic.^55^ Therefore, unlike the other avian influenza subtypes like H1N1, H3N2, new reassortant viruses involving H9N2 viruses are less likely to use swine as the intermediate host/mixing vessel.

In mice and ferrets, we found that the H9N2 viruses infect and replicate asymptomatically, as determined by lung and nasal titers. In the Balb/c mice, only one H9N2 virus (env/Bd/10306) caused weight loss between 3 and 5 d.p.i. without any other clinical signs. When the viral titers of lung samples were initially measured in MDCK cells, they showed very low titer, but when they were measured in eggs, the viral titer was comparable to other H9N2 viruses. This is because the env/Bd/10306 virus possesses the avian host-specific residue (Q226) in the HA to give a lower viral titer in MDCK cells than in eggs.

The quail H9N2 virus (env/Bd/10306) and the chicken ck/BD/10450 virus replicated well in donor ferrets without inducing clinical signs. Two of the three direct-contact ferrets were infected with ck/BD/10450 virus after a delay of four days, and one of the direct contacts shed virus only at a single time point. Although not typically seen in ferret influenza transmission studies, two previous studies^30, 56^ have shown a delayed transmission pattern with H2N2 viruses that acquired the Q226L substitution in HA. Of the two H9N2 isolates tested (env/Bd/10306 and ck/BD/10450), only the chicken H9N2 virus carried the Q226L substitution. This supports the previous conclusion that Q226L substitution has an important role in mammalian transmission.^37^ The observed delay of direct-contact transmission and lack of aerosol transmission suggested that for the H9N2 avian influenza virus to adapt to humans, the virus may require one or many substitutions in addition to the Q226L substitution. Due to logistic limitations, we were unable to undertake a larger ferret study. However, the current results warrant a larger ferret study involving Bangladeshi H9N2 viruses with different molecular markers that have been identified previously.^25^ By doing this, we will be able to correlate and narrow the molecular determinates that are crucial for altering the host range of these viruses.

A number of studies have addressed the molecular basis of host-range specificity or adaptation to new host, but most have focused on individual gene segments such as the HA surface glycoprotein. Pathogenesis, virulence and tropism, however, are multigenic traits.^57, 58, 59^ Several molecular determinants that influence receptor specificity and mammalian-tissue tropism have been identified throughout the Bangladeshi H9N2 genome. It is also interesting to note that the Bangladeshi H9N2 viruses contain three internal genes (*NS*, *PA* and *PB1*) from the HPAI H7N3 virus. This poses an interesting conundrum––despite carrying the molecular markers and genes from the HPAI H7N3, the Bangladeshi H9N2 viruses showed extremely limited mammalian transmission and have caused only one human infection. The viruses' endemic nature and low-virulence profile may have resulted in humans developing widespread immunity to H9N2 due to subclinical infections.^60, 61, 62^

The molecular basis for host range and pathogenesis is poorly understood in H9N2 avian influenza viruses. Rather than using the naturally isolated viruses to gain insight in these areas, we need to use a reverse-genetics approach to generate H9N2 viruses with various combinations of naturally occurring mutations that have been identified in the H9N2 viruses circulating in terrestrial poultry in Bangladesh^25^ and then test those variants in animal models. Using this approach, we can look for features of the continually evolving H9N2 viruses to see if they have reached the optimum gene-constellation level for mammalian infection and transmission and be prepared to intervene if required. However, this approach is currently controversial because it is considered a gain-of-function study. Therefore, the only current recourse is to continuously monitor the H9N2 viruses' evolution, as we have done in this study, and expand that effort.

Monitoring the NAI resistance of influenza viruses is a crucial part of surveillance and risk-assessment studies. NAIs complement vaccines against influenza infections; therefore, emergence of NAI resistance is a serious public health concern. Although all of the H9N2 viruses tested in this study were susceptible to NAIs due to constant reassortment with H5N1, H7N9 and H10N8, the emergence of NAI resistance remains a potential risk. Hence, the continuous monitoring of NAI resistance among H9N2 viruses is essential

The G1-lineage H9N2 avian influenza viruses are of particular concern because they have been implicated in reassortment events with HPAI H5N1 viruses that resulted in human infection;^63^ stand-alone infections in humans, swine and dogs and are also the donor of the internal genes for H7N9 and H10N8 avian influenza viruses infecting humans in China.^1,64^ As a result of the H9N2 vaccination program in China, the G1-clade H9N2 viruses have adapted and evolved tremendously, thereby posing a health concern not only for the poultry industry but also for human health. Because of this, a lot of influenza studies (past and present) involve the Chinese H9N2 viruses and not the Bangladeshi H9N2 viruses. The poultry industry in Bangladesh is very similar to that in China, with live-bird markets in which the human/animal interface is very close. However, China has an H9N2-vaccination strategy, and Bangladesh does not. Our previous study showed that the Bangladeshi H9N2 viruses have a G1-clade origin, but they are very distinct from other G1 viruses (that is, they contain multiple H7N3 internal genes and are evolving constantly).^23^ Our current study shows that the Bangladeshi H9N2 viruses harbor H7N3 internal genes and multiple molecular markers throughout their genome. They also possess the potential to induce subclinical infections in ferrets but do not pose a threat to human health currently because they lack transmission potential. This finding is supported by the fact that no other mammalian H9N2 infection has been identified in Bangladesh, with the exception of a single human infection in 2011. This is extremely important, considering how prevalent H9N2 is in Bangladesh and how close the animal/human interface is. Our results suggest that the *H9N2* gene constellation is currently not optimized. This emphasizes the need for further studies that monitor the viruses' evolution and aim to increase our understanding of the transmission and potential risk that these H9N2 viruses pose to humans.

## Figures and Tables

**Table 1:** Bangladeshi H9N2 influenza viruses used in this study

**H9N2 avian influenza virus**	**Abbreviation**
A/environment/Bangladesh/8202/2010 (chicken)^a^	env/Bd/8202
A/chicken/Bangladesh/8996/2010	ck/Bd/8996
A/environment/Bangladesh/10306/2011 (quail)^a^	env/Bd/10306
A/chicken/Bangladesh/10450/2011	ck/Bd/10450
A/chicken/Bangladesh/10897/2011	ck/Bd/10897
A/chicken/Bangladesh/11173/2011	ck/Bd/11173
A/environment/Bangladesh/16777/2012 (quail)^a^	env/Bd/16777
A/environment/Bangladesh/17403/2012 (quail)^a^	env/Bd//17403
A/quail/Bangladesh/19462/13	qa/Bd/19462

a The virus was isolated from the cages of chicken or quail.

**Table 2:**
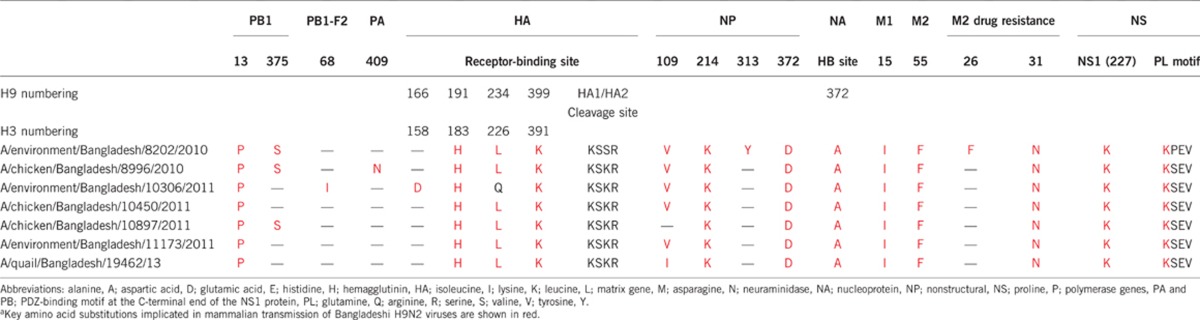
Mammalian host–tropic mutations^a^ in the surface glycoproteins (HA and NA) and internal proteins of Bangladeshi H9N2 influenza viruses used in this study

**Table 3:** Replication of Bangladeshi H9N2 influenza A viruses in juvenile swine explants

**Bangladeshi H9N2 viruses and controls**	**Virus yield in tracheal explants TCID**_**50**_ **(log**_**10**_**/mL)**				**Virus yield in lung explants TCID**_**50**_ **(log**_**10**_**/mL)**			
	**2 h**	**24 h**	**48 h**	**72 h**	**2 h**	**24 h**	**48 h**	**72 h**
A/env/Bangladesh/8202/2010 (chicken)^a^	<	1.0	1.0	1.0	<	2.4	5.5	5.3
A/chicken/Bangladesh/8996/2010	<	2.3	1.0	1.0	<	3.0	4.6	4.3
A/env/Bangladesh/10306/2011 (quail)^a^	<	<	<	<	<	<	3.5	4.5
A/chicken/Bangladesh/10450/2011	<	3.5	3.8	4.3	<	3.5	4.5	4.5
A/env/Bangladesh/16777/2012 (quail)^a^	<	2.5	1.0	2.5	<	2.8	5.3	5.5
A/env/Bangladesh/17403/2012 (quail)^a^	<	2.5	2.4	2.4	<	<	4.3	4.5
A/chicken/Bangladesh/11173/2011	<	3.5	3.8	4.5	<	4.8	5.5	5.5
A/swine/Missouri/2124514/2006 (H2N3)^b^	1.0	3.4	6.3	5.5	2.5	4.8	4.8	4.5
A/duck/New Jersey/872-27/78 (H2N2)^c^	<	<	<	<	<	<	<	<

Abbreviation: tissue culture infectious dose, TCID.

a Virus was isolated from the cages of chickens or quail.

b Mammalian control virus.

c Avian control virus. < sign indicates that the virus was not detected at concentration less than 1 log10/mL and is less than the limit of detection.

**Table 4:** Viral and hemagglutination inhibition titers in Balb/c mice infected with Bangladeshi H9N2 viruses

**Bangladeshi H9N2 virus**	**Lung TCID**_**50**_ **(log**_**10**_**/mL)**		**Number of mice positive for HI/total number of mice (HI titer range)**
	**3 d.p.i.**	**5 d.p.i.**	
A/env/Bangladesh/8202/2010 (chicken)	5.1	3.3	5/5 (320–1280)
A/chicken/Bangladesh/8996/2010	4.9	4.9	5/5 (160–320)
A/env/Bangladesh/10306/2011 (quail)	1.0	1.0	4/5 (40–160)
A/chicken/Bangladesh/10450/2011	5.6	4.7	5/5 (160–640)
A/chicken/Bangladesh/10897/2011	5.4	3.6	5/5 (80–320)
A/quail/Bangladesh/19462/2013	3.85	3.0	5/5 (80–320)

Abbreviations: days post infectoion, d.p.i.; tissue culture infectious dose, TCID.

**Table 5:** Replication and transmission of Bangladeshi H9N2 viruses in ferrets^a^

**Bangladeshi H9N2 viruses**	**Ferret #**	**Sneeze TCID**_**50**_ **(log**_**10**_**/mL)**					**HI titer**
		**2 d.p.i.**	**4 d.p.i.**	**6 d.p.i.**	**8 d.p.i.**	**10 d.p.i.**	
**A/chicken/Pakistan(NARC)/1624/2005**							
Control	1	5.5	5.0	6.5	3.3		1280
	2	6.5	5.3	6.0	<	<	2560
	3	6.3	3.0	6.2	<		640
							
**A/env/Bangladesh/10306/ 2011(quail)**							
Donor	1	4.3	<	6.0	<		160
	2	3.5	2.7	2.5	<	<	160
	3	4.5	3.5	3.2	<		160
DC	1			<			<10
	2						<10
	3						<10
AC^b^	1			<			<10
	2						<10
	3						<10
							
**A/chicken/Bangladesh/10450/ 2011**							
Donor	1	5.3	4.8	5.0			1280
	2	6.2	5.5	5.5	5.6	<	1280
	3	6.3	6.5	5.8			1280
DC	1	<	<	<	<		<10
	2	<	2.5	2.8	4.0	<	640
	3	<	<	5.8	<		1280
AC	1			<			<10
	2						<10
	3						<10

Abbreviation: tissue culture infectious dose, TCID.

a Each virus was tested in nine ferrets: three donors, three direct-contact (DC) ferrets, and three aerosol-contact (AC) ferrets.

b None of the aerosol contact for env/BD/10306 had detectable viral titers and did not seroconvert at 14 d.p.i.

b < sign indicates that the virus was not detected at concentration less than 1 log10/ml and is less than the limit of detection.

**Table 6:** Susceptibility of Bangladeshi H9N2 influenza viruses to neuraminidase inhibitors

**Virus**	**Inhibitory activity (IC**_**50**_**±SD, nM)**^a^		
	**Oseltamivir**	**Zanamivir**	**Peramivir**
A/env/Bd/8202/10 (H9N2)	0.14±0.01	0.56±0.04	0.16±0.00
A/ck/Bd/8996/10 (H9N2)	0.20±0.03	0.69±0.15	0.21±0.02
A/env/Bd/10306/11 (H9N2)	0.14±0.02	0.49±0.06	0.13±0.01
A/ck/Bd/10450/11 (H9N2)	0.14±0.03	0.63±0.14	0.17±0.01
A/env/Bd /17403/12 (H9N2)	0.20±0.05	0.63±0.05	0.21±0.03
A/ck/Bd/11173/11 (H9N2)	0.13±0.02	0.43±0.03	0.13±0.01
A/Fuk/20/04 WT (H3N2)	0.27±0.00	0.78±0.01	0.23±0.02
A/Fuk/45/04^b^ (E119V) (H3N2)	69.07±11.45	1.12±0.08	0.25±0.00

a Mean inhibition of NA enzymatic activity with MUNANA substrate (100 μM final concentration).

b Neuraminidase inhibitor–resistant virus.
